# Complement Inhibition for Geographic Atrophy: A Tempting Target with Mixed Results

**DOI:** 10.3390/jcm10132890

**Published:** 2021-06-29

**Authors:** Jonathan B. Lin, Omar A. Halawa, Joan W. Miller, Demetrios G. Vavvas

**Affiliations:** Department of Ophthalmology, Massachusetts Eye and Ear and Harvard Medical School, Boston, MA 02114, USA; jblin6@gmail.com (J.B.L.); omar_halawa@hms.harvard.edu (O.A.H.)

**Keywords:** age-related macular degeneration, geographic atrophy, complement, retina, drusen

## Abstract

Age-related macular degeneration (AMD) is a leading cause of blindness in older adults. One of the strongest genetic risk factors for AMD is a complement factor H (CFH) gene polymorphism characterized by a tyrosine-histidine change at amino acid position 402 (Y402H). The magnitude of this association between the Y402H variant and AMD is among the strongest that has been identified for any complex, multifactorial human disease. This strong association has motivated researchers to investigate a potential link between various elements of the complement pathway and AMD pathogenesis. Given the possible contribution of complement dysregulation to AMD, complement inhibition has emerged as a therapeutic strategy for slowing geographic atrophy (GA). Randomized clinical trials thus far have yielded mixed results. In this article, we provide the historical context for complement inhibition as a strategy for treating GA, discuss potential advantages and disadvantages of complement inhibition, and highlight the questions that must be addressed before complement inhibition can take center stage as a therapy for AMD.

## 1. Introduction

Age-related macular degeneration (AMD) is a leading cause of blindness in older adults and is projected to affect as many as 288 million people by 2040 [1]. Advanced AMD manifests in two forms: non-exudative (dry) AMD and exudative (wet) AMD. Advanced nonexudative AMD is characterized by degeneration of photoreceptors, retinal pigment epithelium (RPE) cells, and choriocapillaris, termed geographic atrophy (GA); advanced exudative AMD is characterized by abnormal blood vessel growth beneath the retina. When left untreated, both forms of advanced AMD can progress to irreversible vision loss and devastating blindness.

Although exudative AMD was historically responsible for the majority of vision loss associated with AMD, the development of therapies targeted against vascular endothelial growth factor (VEGF) has been transformative. Anti-VEGF medications such as bevacizumab, ranibizumab, aflibercept, and, most recently, brolucizumab, can be delivered into the intravitreal space and reduce the extent of exudative AMD. Anti-VEGF agents are not a panacea since some patients do not respond or under-respond for unclear reasons [2]. Nonetheless, they have improved visual outcomes for many patients with exudative AMD, although some do develop macular atrophy after prolonged anti-VEGF therapy.

Despite advances in our ability to manage patients with exudative AMD, we are unequipped to treat GA as seen in nonexudative AMD. There are no approved treatments that prevent or slow progression of GA. Although vitamin and antioxidant supplementation may reduce the risk of progression to advanced AMD, they appear to reduce the risk of progression specifically to exudative AMD and may, in fact, have no effect on either risk of developing GA or rate of GA lesion growth [3,4,5,6,7]. There is a clinical need for novel therapies for GA. Given a potential link between the complement system and AMD, complement inhibition has emerged as a strategy for treating GA.

## 2. Evidence of Complement Dysregulation in AMD Pathogenesis

**Figure 1:**
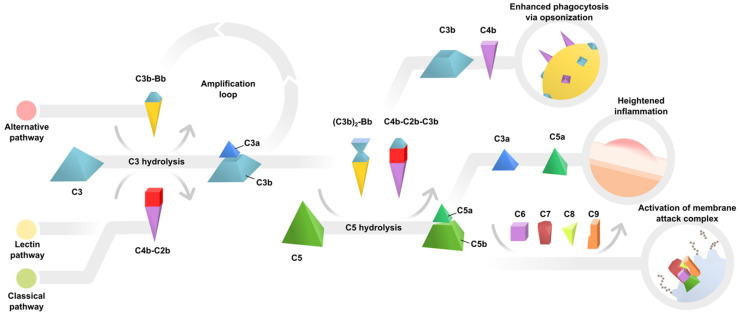
Schematic detailing key effectors of the complement pathway.

The complement system is an integral and ancient part of the immune system. The complement cascade is activated by three well-characterized pathways: the classical complement pathway, the lectin complement pathway, and the alternative complement pathway. Each of these initiating pathways forms a C3 convertase (C4b-C2b or C3b-Bb), an enzyme that catalyzes the hydrolysis of C3 to C3a/C3b and thereby activates the common final pathway of the complement cascade. The effect of common final pathway activation is threefold: (1) enhanced phagocytosis of foreign or damaged material; (2) heightened inflammation; and (3) activation of the pathogen-killing membrane attack complex (MAC) (Figure 1). Numerous soluble and membrane-associated complement regulators prevent inappropriate complement activation. The complex pathways involved in complement activation and complement regulation are discussed in excellent reviews [8,9].

The initial evidence suggesting that the complement pathway may be involved in AMD pathogenesis stemmed from landmark genetic studies published at the turn of the millennium. Specifically, genome-wide association studies identified that a tyrosine-histidine change at amino acid position 402 (Y402H) in the complement factor H (CFH) gene was associated with a greater than 2 to 3-fold increased odds of AMD [10,11,12]. The magnitude of this association is among the strongest that has been identified for any complex, multifactorial disease to date. When it was initially identified, it was thought that the Y402H variant may account for up to 50% of the attributable risk of AMD [12].

CFH is a negative regulator of the alternative pathway of complement activation. The Y402H variant is located within the short consensus repeat 7 (SCR7) region of the CFH gene and affects the ability of CFH to bind to molecules such as heparin, C-reactive protein, and oxidized phospholipids [13,14,15,16]. Although the exact mechanism is an area of active investigation, it is thought that alteration in CFH’s binding kinetics confers increased risk of AMD by impairing its ability to negatively regulate alternative pathway activation.

Since these seminal studies, other complement gene variants, including those in complement factor I (CFI), complement factor D (CFD), complement factor B (CFB), and complement component 2 (C2), among others, have been found to be associated with increased odds of AMD [17,18,19]. Moreover, complement activation products have been identified within drusen, lipid- and protein-rich deposits that develop in patients with AMD [20,21,22]. Patients with AMD also have higher systemic complement activation compared to non-AMD controls [23]. These findings provide evidence that complement dysregulation is involved in AMD pathogenesis.

## 3. Complement Inhibition for Geographic Atrophy

Given this possible relationship between complement dysregulation and AMD, numerous clinical trials have evaluated complement inhibition as a strategy for treating GA. This section describes completed Phase 2 and 3 trials that were found on clinicaltrials.gov (accessed on 15 January 2021).

### 3.1. Studies Reporting Null Results

The initial studies yielded null results (Table 1). In the Phase 2 COMPLETE study, intravenous administration of the anti-C5 humanized antibody eculizumab (Soliris^®^; Alexion Pharmaceuticals, Boston, MA, USA) had no effect on GA growth rate [24]. Similarly, intravitreal administration of the anti-C5 fully human antibody LFG316 (Novartis, Basel, Switzerland) did not significantly change GA growth rate or visual acuity (NCT01527500). Although the anti-CFD antibody lampalizumab (Genentech, San Francisco, CA, USA) met a pre-specified alpha of 0.20 in the Phase 2 MAHALO study, the Phase 3 SPECTRI (NCT02247531) and CHROMA (NCT02247479) studies showed no effect of lampalizumab on GA growth rate [25,26]. Despite initial promise, these null results suggest that complement inhibition may not be the appropriate strategy for treating GA.

### 3.2. Studies Reporting Statistically Significant Results

In the Phase 2 FILLY study, intravitreal injection of the C3 inhibitor pegcetacoplan (APL-2; Apellis Pharmaceuticals; Crestwood, KY, USA) significantly reduced GA growth rate [27]. In the Phase 2/3 GATHER1 study, intravitreal injections of the anti-C5 aptamer avacincaptad pegol/ARC1905 (Zimura^®^; IVERIC Bio [formerly, Ophthotech], Cranbury, NJ, USA) significantly reduced GA growth rate [28]. Phase 3 studies (ARC1905: GATHER2 [NCT04435366]; APL-2: DERBY [NCT03525613] and OAKS [NCT03525600]) are ongoing. A summary of the results of all mentioned studies is presented in Table 1.

### 3.3. Some Notable Observations

**Figure 2:**
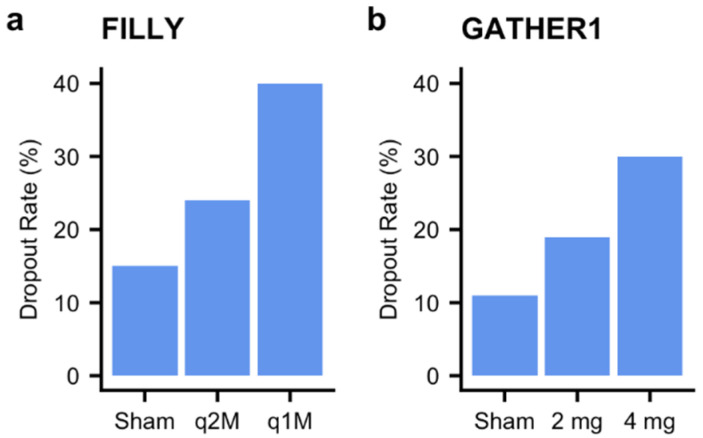
Increased drop-out rates in the treatment arms of complement inhibition studies. Dropout rates in the sham and treatment arms of the (**a**) FILLY [27] and (**b**) GATHER1 [28] studies. q2M: treatment every 2 months; q1M: treatment every 1 month.

Despite meeting their primary endpoint, the FILLY and GATHER1 studies have notable features that warrant a closer look. First, in both studies, there was a high dropout rate of up to 30–40% among patients who were randomized to the treatment arm compared to 10–15% among patients who were randomized to the placebo arm (Figure 2). Although no patients dropped out of the COMPLETE study, there were similarly high drop-out rates of up to 25–30% in the treatment arms of the Phase 2 MAHALO study versus 17% in the pooled sham arm. In contrast, all arms of the Phase 3 CHROMA/SPECTRI trials had a drop-out rate of less than 10%. The specific reasons for dropping out need to be examined closely to determine whether they may affect the ability of complement inhibition to become widely used for patients with GA. Furthermore, the specific analytic approaches used to handle this dropout are not clearly delineated in the published manuscripts and thus requires further scrutiny to determine whether they could contribute to potential bias. Commonly used methods for this type of analysis, such as mixed repeated-measures models, rely on the assumption that missing data is missing at random. If the missing data was not at random (i.e., there was a difference in patients who dropped out versus those who remained in the study), the significant findings could have been influenced by bias. Sensitivity analyses performed by the investigators suggested that these concerns may not be of significance but are still worth closer examination given that they may lead to a new therapy for GA.

**Figure 3:**
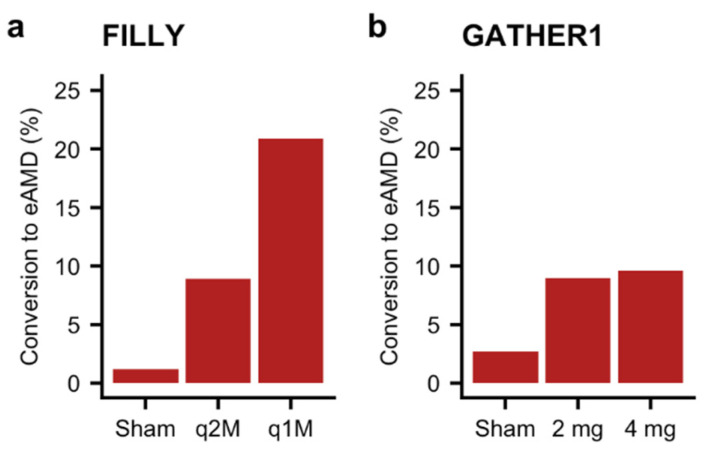
Increased conversion to exudative AMD with complement inhibition. Rates of conversion to exudative age-related macular degeneration (eAMD) in the sham and treatment arms of the (**a**) FILLY [27] and (**b**) GATHER1 [28] studies. q2M/q1M: treatment every 2 months or 1 month.

Second, patients in the FILLY and GATHER1 studies who were randomized to intravitreal complement inhibition were 3 to 17 times more likely to develop exudative AMD compared to those who received sham injections [27,28] (Figure 3). Conversion to exudative AMD not only may hinder precise quantification of GA area but is also potentially concerning in light of some animal studies that support that complement inhibition may promote more extensive neovascularization in a laser-injury model [29], although not all animal studies have yielded the same results. Post hoc analysis of the FILLY study suggests that development of exudative AMD was more likely in patients with risk factors at baseline, such as presence of the double-layer sign on imaging in the study eye or history of exudative AMD in the contralateral eye, suggesting that complement inhibition may have the unintended consequence of increasing risk of exudation in patients who have underlying risk factors [30]. Finally, the FILLY study reported a 2% rate of endophthalmitis in patients who were randomized to monthly intravitreal APL-2 injections. Given that complement inhibition impairs the immune system, this risk is biologically plausible and requires careful scrutiny given the potentially devastating and immediate visual consequences of endophthalmitis.

Chronic complement inhibition may also have biological consequences beyond the 12 to 18-month follow-up period that has been studied. Animal studies suggest that the complement pathway may be important for maintaining retinal homeostasis and resilience against retinal neurodegeneration. Yu and colleagues reported that mice lacking C3a- and C5a-mediated signaling exhibited progressive retinal degeneration compared to wild-type controls [31]. Similarly, other studies have identified that complement proteins are important for maintaining retinal integrity in aging and other models of retinal disease [32,33,34]. The key findings from these selected studies are shown in Table 2. If also true in humans, aggressive complement inhibition may have unintended, long-term consequences. A detailed description of other animal models that highlight the importance of the complement pathway in ocular phenotypes is discussed in other reviews [35,36].

### 3.4. The Right Drug for the Wrong Patient?

**Figure 4:**
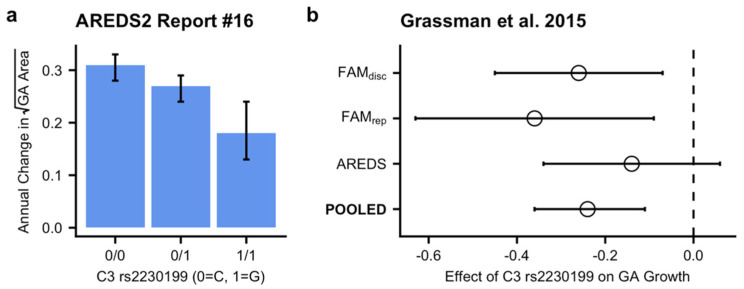
Decreased geographic atrophy (GA) progression with C3 risk allele. (**a**) Annual change in square root-transformed GA area stratified by presence of risk-conferring C3 alleles as reported in AREDS2 Report #16 [41]. (**b**) Meta-analyzed effect of C3 rs2230199 risk allele on GA growth from Fundus Autofluorescence in Age-Related Macular Degeneration (FAM study; disc: discovery cohort; rep: replication cohort) and Age-Related Eye Disease Study (AREDS) with the vertical dashed line showing the null result of no statistically significant effect. (**a**,**b**) Error bars indicate 95% confidence intervals.

The magnitude of association between complement gene variants and AMD is among the strongest that has been identified for any complex, multifactorial disease. A recent study by Heesterbeek and colleagues revealed a small but statistically significant increase in serum C3d/C3 ratios, a marker of complement activation, in patients with intermediate AMD or central GA versus patients without AMD [23], supporting that chronic, low systemic complement dysregulation may contribute to AMD. However, this study did not investigate whether there is an association between complement activation and GA growth rate. While many studies have found a strong association between complement gene variants and the presence of AMD, there is not a clear association between risk-conferring gene variants and AMD progression [37,38,39]. Secondary analysis of the COMPLETE study revealed no significant associations between risk-conferring complement gene variants and either baseline GA area or GA growth rate [24], although this finding should be interpreted cautiously since it was not the primary aim of the study. Certain variants of C3 have even been shown to be associated with an increased risk of AMD but a lower GA growth rate [40,41] (Figure 4).

These discrepancies suggest that there is a complex relationship between complement dysregulation and AMD pathogenesis that requires further investigation. Studies that analyze complement activation profiles at specific, well-defined stages of AMD could provide further insight into the role of complement dysregulation in various stages of AMD. If supported by the data, it is plausible that complement inhibition may have a role for treating patients who are at risk of developing AMD rather than for preventing progression to advanced disease, although the potential risks of regular intravitreal injections may not outweigh the potential benefits of complement inhibition for prevention of AMD.

### 3.5. Should We Pause or Proceed?

There is clear evidence that the complement pathway is involved in the pathogenesis of AMD, although the cellular and molecular mechanisms underlying this complex relationship are still an area of active investigation. Despite these limitations in our understanding in the underlying pathobiology, significant resources have been invested in clinical trials that evaluate complement inhibition as a strategy to slow GA progression. To date, no Phase 3 trial has met its primary endpoint of showing an effect of complement inhibition on GA growth; nonetheless, many agents remain under active investigation. A summary of the evidence for and against complement inhibition as a viable strategy for treating GA is presented in Table 3.

We should proceed with caution as we continue to investigate new therapeutic strategies for GA. Although discovering a treatment for GA remains a priority given the lack of treatments available at this time, there are disadvantages to pursuing strategies that have, thus far, been unsuccessful. Continued investigations into the pathobiology of AMD that offer insights into how complement gene variants lead to increased risk of AMD and characterize what specific portions of the complement pathway become dysregulated at a local and systemic level are necessary. These findings may inform the design of clinical trials that target the optimal patient population with the optimal agent.

The complement pathway is complex with numerous levels of regulation. A better understanding of the specific effectors that contribute to AMD pathophysiology may lead to pathway-specific therapies rather than broad inhibitory strategies. These targeted approaches may slow AMD progression with fewer off-target, unintended effects. Given that there are reports that certain genotypes are associated with distinct responses to current AMD treatments, it may also be important to investigate whether patients with measurable complement dysregulation, whether due to underlying genetics or otherwise, may be more responsive to complement modulation [6,42,43]. There have also been other pathways, such as oxidative stress and metabolic dysfunction, that have been proposed to contribute to photoreceptor and RPE degeneration as observed in GA [44,45,46,47]. Although many of these hypotheses are based primarily on animal studies, it will be important to elucidate how these pathologic processes interact with complement dysregulation to be able to develop therapies for GA that target the underlying primary pathologic process.

## 4. Conclusions

Despite excitement surrounding the use of complement inhibition to treat GA, there are numerous questions that need to be answered. Additional research that identifies the disease stage of AMD that would benefit the most from complement inhibition, rigorously evaluates possible unintended consequences of complement inhibition, and addresses potential tolerability concerns is necessary. A new therapy that would revolutionize the therapeutic landscape for patients with GA necessitates a better understanding of disease pathophysiology and critical assessment of all available data.

## Figures and Tables

**Table 1:** Published Phase 2 and 3 clinical trials evaluating complement inhibition to slow geographic atrophy (GA).

Study (Phase)	Drug (Target)	Group 1	Group 2	Sham		Group 1			Group 2			Time ^b^
				N	GA Growth	N	GA Growth	P ^a^	N	GA Growth	P ^a^	
COMPLETE (2)	Eculizumab (C5)	Low dose	High dose	10	0.37 ^c^	10	0.35 ^c^	N.S.	10	0.40 ^c^	N.S.	52 W
MAHALO (2)	Lampalizumab (CFD)	q2M	q1M	40	0.30 [2.92] ^d^	41	0.33 [3.15] ^d^	0.552	42	0.25 [2.33] ^d^	0.117	18 M
CHROMA/SPECTRI (3)	Lampalizumab (CFD)	q6W	q4W	598	0.36 [1.98] ^d^	603	0.36 [2.05] ^d^	N.S.	596	0.37 [2.06] ^d^	N.S.	48 W
FILLY (2)	APL-2 (C3)	q2M	q1M	81	0.35 ^c^	79	0.28 ^c^	0.067	86	0.26 ^c^	0.008	12 M
GATHER1 (2/3)	ARC1905 (C5)	Low dose	High dose	194	0.42 ^e^	67	0.29 ^c^	0.007	83	0.32 ^c^	0.005	12 M

^a^ *p*-value as reported in published study, N.S.: not significant; ^b^ Follow-up duration, W: weeks, M: months; ^c^ Adjusted mean change in square root-transformed geographic atrophy (GA) area; ^d^ When studies reported adjusted mean change in untransformed GA area, we calculated an approximation of the square-root transformation for ease of comparison by finding the difference between the square root-transformed baseline GA area and square root-transformed final GA area and dividing the difference by the follow-up duration in years (the original untransformed values are shown in brackets); ^e^ Weighted average of multiple sham groups.

**Table 2:** Selected basic science studies identifying key role of complement in retinal homeostasis in mouse models.

Study Authors	Year	Key Finding
Yu et al. [31]	2012	Mice lacking receptors for C3a and C5a develop retinal degeneration
Hoh Kam et al. [32]	2013	Mice lacking C3, CFH, or both develop retinal degeneration during aging
Mukai et al. [33]	2018	Mice lacking C1q, Mbl, Fb, C3, and C5 have accelerated retinal degeneration with aging
Silverman et al. [34]	2019	C3-CR3 signaling protects against degeneration in a mouse model of retinitis pigmentosa

**Table 3:** Evidence for and against complement inhibition as a viable strategy for slowing geographic atrophy.

Supporting Evidence	Opposing Evidence
Magnitude of association between complement genes and AMD is among the strongest reported for any complex, multifactorial disease	No proven genetic association of complement with GA progression with some studies showing that certain C3 variants may be associated with slower GA progression
Some Phase 2 studies have shown sufficient promise to proceed to Phase 3 trials that are currently ongoing (GATHER2, DERBY, OAKS)	Phase 2 with promising results have high dropout rates in the treatment arm and increased conversion to exudative AMD, requiring further exploration
Evidence of both systemic and local complement over-activation in patients with AMD supports that complement inhibition may be beneficial	Complement inhibition may have unintended consequences in terms of conversion to exudative AMD, increased risk of endophthalmitis, or damaging retinal health in the long-term time frame

## Data Availability

All reported results are from previously published studies.
